# A Novel Statistical Method to Diagnose, Quantify and Correct Batch Effects in Genomic Studies

**DOI:** 10.1038/s41598-017-11110-6

**Published:** 2017-09-07

**Authors:** Gift Nyamundanda, Pawan Poudel, Yatish Patil, Anguraj Sadanandam

**Affiliations:** 1 Division of Molecular Pathology, The Institute of Cancer Research, London, United Kingdom; 2 Centre for Molecular Pathology, Royal Marsden Hospital, London, United Kingdom

## Abstract

Genome projects now generate large-scale data often produced at various time points by different laboratories using multiple platforms. This increases the potential for batch effects. Currently there are several batch evaluation methods like principal component analysis (PCA; mostly based on visual inspection), and sometimes they fail to reveal all of the underlying batch effects. These methods can also lead to the risk of unintentionally correcting biologically interesting factors attributed to batch effects. Here we propose a novel statistical method, finding batch effect (*findBATCH*), to evaluate batch effect based on probabilistic principal component and covariates analysis (PPCCA). The same framework also provides a new approach to batch correction, correcting batch effect (*correctBATCH*), which we have shown to be a better approach to traditional PCA-based correction. We demonstrate the utility of these methods using two different examples (breast and colorectal cancers) by merging gene expression data from different studies after diagnosing and correcting for batch effects and retaining the biological effects. These methods, along with conventional visual inspection-based PCA, are available as a part of an R package exploring batch effect (*exploBATCH*; https://github.com/syspremed/exploBATCH).

## Introduction

Batch effect refers to technical variation or non-biological differences between measurements of different groups of samples. Although batch effect can be reduced by good experimental design, it is difficult to completely eradicate^1^. If this systematic bias is not removed, its effect can mask important biological differences (discussed in results section using colorectal cancer as an example), at worst resulting in misleading inferences and conclusions.

Many approaches have now been developed to remove batch effects from high-throughput genomic profiling datasets. Common methods include: combating batch effect (*ComBat*), an empirical Bayes method for batch correction on each gene^2^; distance-weighted discrimination (*DWD*), which employs support vector machines (SVMs) to find a hyper-plane separating the batches^3^ (both *ComBat* and *DWD* were used by us in multiple instances^4–7^); *FAbatch*, an extension of *ComBat* with batch-specific latent variables that is only suitable when the outcome of interest is known and binary^8^; mean-adjustment of microarray data by batches using prediction analysis of microarrays - *PAM* ^9^; gene standardisation by *z-score* ^10^; cross-platform normalisation (*XPN*), which is based on fitting a block linear model on clusters of features and samples from the different datasets to be merged^11^; and finally, PCA/singular value decomposition (SVD), which searches for directions of maximal variance associated with batch effect in the data space and removes them^12, 13^. The main drawback of PCA is that, if batch effect is not the greatest source of variability PCA fails as a batch correction method^3^. In addition, we are going to show that correcting batch effect by completely removing principal components (PCs) affected by batch can result in loss of essential non-technical information, as this variability may not be exclusively due to batch effect. At present *ComBat* is the standard method and it has been shown to outperform most of the available batch correction approaches^1^. Therefore, we have used *ComBat* to compare to our *exploBATCH* method.

Most batch effect studies focus on methods to remove systematic bias in high-throughput genomic data rather than on tools to detect, evaluate, or diagnose batch effect before and after correction. The current standard methods for detecting batch effect including PCA, dendrograms, boxplots, and density plots are based on visualisation and can only be regarded as explorative in nature^10^. PCA is the most standard approach in this setting and is based on visual inspection of the first few PCs^10, 14^. These *ad hoc* approaches can be subjective and, when within-batch variability is high relative to total batch variability, PCA usually provides inconclusive evidence of the presence of batch effect^3, 14^. Moreover, unnecessary batch correction can lead to unwarranted data distortion^10^.

There are few metrics available to investigate batch effect including: *a)* the mixture score, which uses a *k*-nearest neighbour-based distance metric to assess how samples from different batches mix^15^; *b)* the skewness divergence score (*skewdiv*), which measures the distributional differences between data from different batches^8^; *c)* average minimal distance to the other batch (*avedist*) which uses Euclidean distances to measure separation between batches^8^; and *d)* the Kullback-Leibler divergence score (*klmetr*), which assesses variability within and between batches^16^. However, none of these methods provide a formal statistical test to evaluate the presence of batch effect(s) in the data.

Principal variation component analysis (*PVCA*) is another batch evaluation method that identifies sources of variability in data^1, 17^. Specifically, PVCA is a multi-step method that initially reduces data dimensionality using PCA followed by estimating the variability associated with batches using a linear mixed model fitted on each PC^1, 17^. Finally, PVCA derives the proportion of variability associated with batch effect using the estimated batch variability from the linear mixed model and eigenvalues associated with each PC from PCA^1^. Although this method has been successfully applied to compare the performances of different batch correction methods, it has the following main limitations in diagnosing batch effects: (i) it involves multiple batch evaluation steps, which reduces statistical power; (ii) there is no standard approach for selecting the optimal number of PCs associated with the data; and (iii) it does not use a formal statistical test to assess the significance of the batch effects. Hence, there remains a need for methods that perform formal statistical testing to significantly evaluate/diagnose the batch effect(s) before and after batch correction.

Here we propose a new batch evaluation and correction approach called explore batch effect (*exploBATCH*) based on PPCCA, which we originally developed to discover metabolites associated with cancer phenotypes^18^. Since the PPCCA framework allows for incorporation of covariates into traditional PCA, *findBATCH* (within *exploBATCH*) employs PPCCA to evaluate and detect the presence of significant batch effects by statistically testing if the samples are distributed according to batches in the principal subspace. Reese *et al*. developed guided PCA (*gPCA*), an extension of traditional PCA, to discover batch effects in high-throughput genomic data^14^. However, although *gPCA* provides a permutation-based formal statistical test of batch effect, it is a global test on all the PCs. Instead, *findBATCH* statistically tests every PC for the presence of batch effect. Furthermore, we have developed a new method *correctBATCH* (a part of *exploBATCH* and again based on PPCCA) for batch correction that subtracts the batch effect on each affected PC to recover the batch-corrected expression data. We evaluate this package using examples from breast (GSE12763^19^, GSE13787^20^ and GSE23593^21^) and colorectal cancer (CRC; GSE18088^22^ and GSE23878^23^) and normal sample gene expression profiles.

## Results and Discussion

### Framework *of exploBATCH* for batch detection and correction

**Figure 1:**
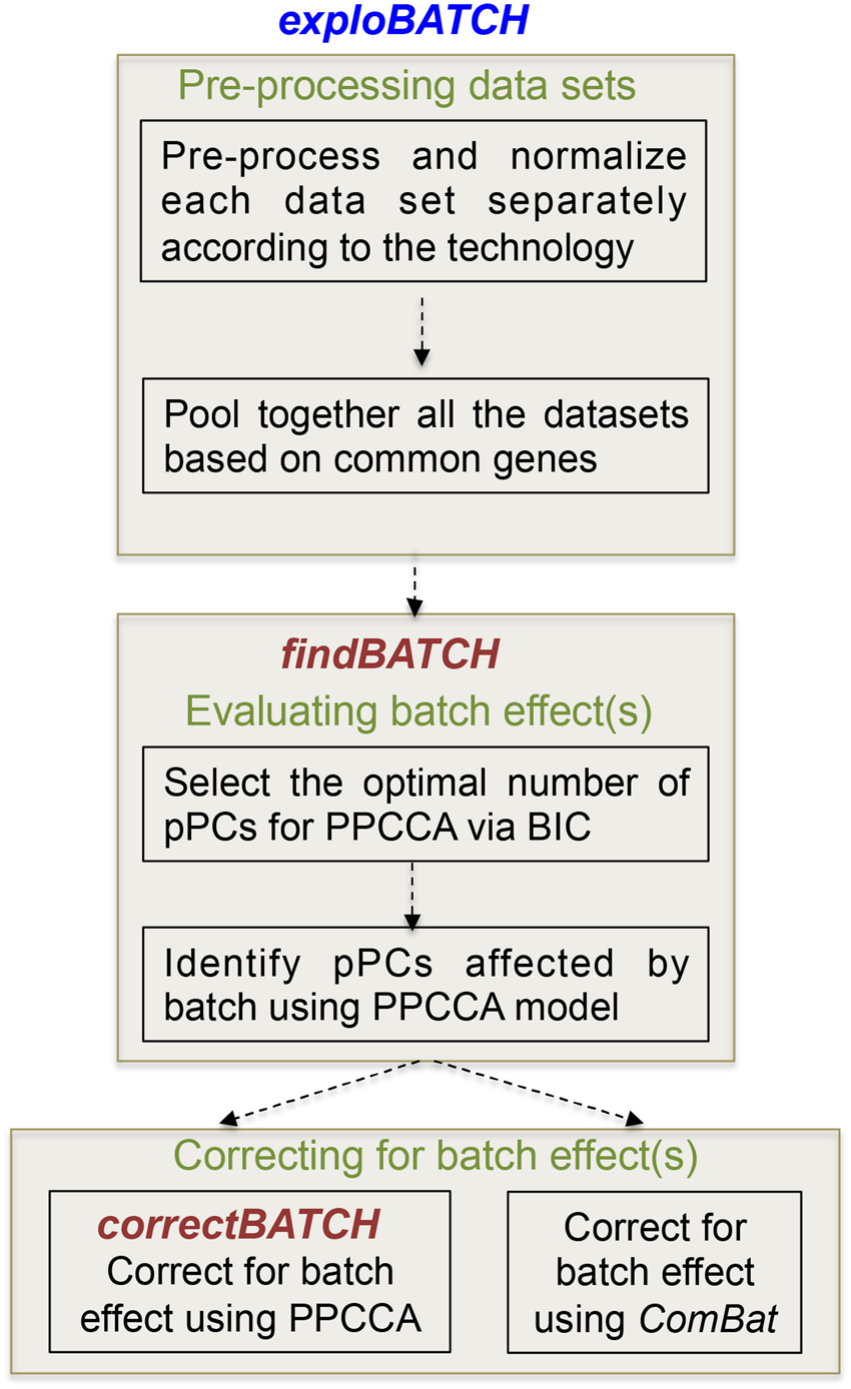
Flowchart of steps within *exploBATCH*. A schematic representation of the steps involved within *exploBATCH* for batch detection, quantitation and correction.

The main challenge in merging different datasets is detecting and correcting for systematic bias (due to the fact that data are generated from different sources, time points or platforms) without distorting important biological effects. The different steps involved in evaluating batch effect(s) in *exploBATCH* are illustrated in Fig. 1. First, each individual dataset is separately pre-processed and normalised according to the technology used and then pooled together based on common identifiers (probes or gene names). Second, *findBATCH* within *exploBATCH* is used to evaluate the existence of batch effect(s) in the data. The *findBATCH* function selects the optimal number of probabilistic (p)PCs (based on the highest Bayesian information criterion value; BIC; Nyamundanda *et al*., 2010)^18^ associated with PPCCA and exploits variability associated with the batch variable to quantify and test the effect of batch(es) in the data. The *findBATCH* function computes 95% confidence intervals (CI) around the estimated batch effect on each pPC. Those pPCs with 95% CI values not including zero are considered to have significant batch effect. Finally, if one or more batch effects exist, *correctBATCH* subtracts the effect of batch on each affected probabilistic principal component (pPC) to recover the batch-corrected expression data. *ComBat* (standard approach for batch correction in genomics^1^) was included as part of *exploBATCH*, however, any other batch correction method can be implemented within *exploBATCH* when required. Both *findBATCH* and *correctBATCH* are implemented in ***R*** statistical software as *exploBATCH*. The *exploBATCH* output includes: (i) Forest plots, showing estimated batch effect(s) with corresponding 95% CIs to identify pPCs significantly associated with batch; (ii) PCA and PPCCA plots before and after batch correction for visual inspection; and (iii) batch-corrected expression data using *correctBATCH* or *ComBat*. Overall, *exploBATCH* provides a framework for formal statistical testing to assess and quantitate the batch effect(s), which also allows for batch correction.

### Demonstration of *exploBATCH*

#### Detecting, quantitating and correcting for batch effect - merging three breast cancer gene expression datasets

**Figure 2:**
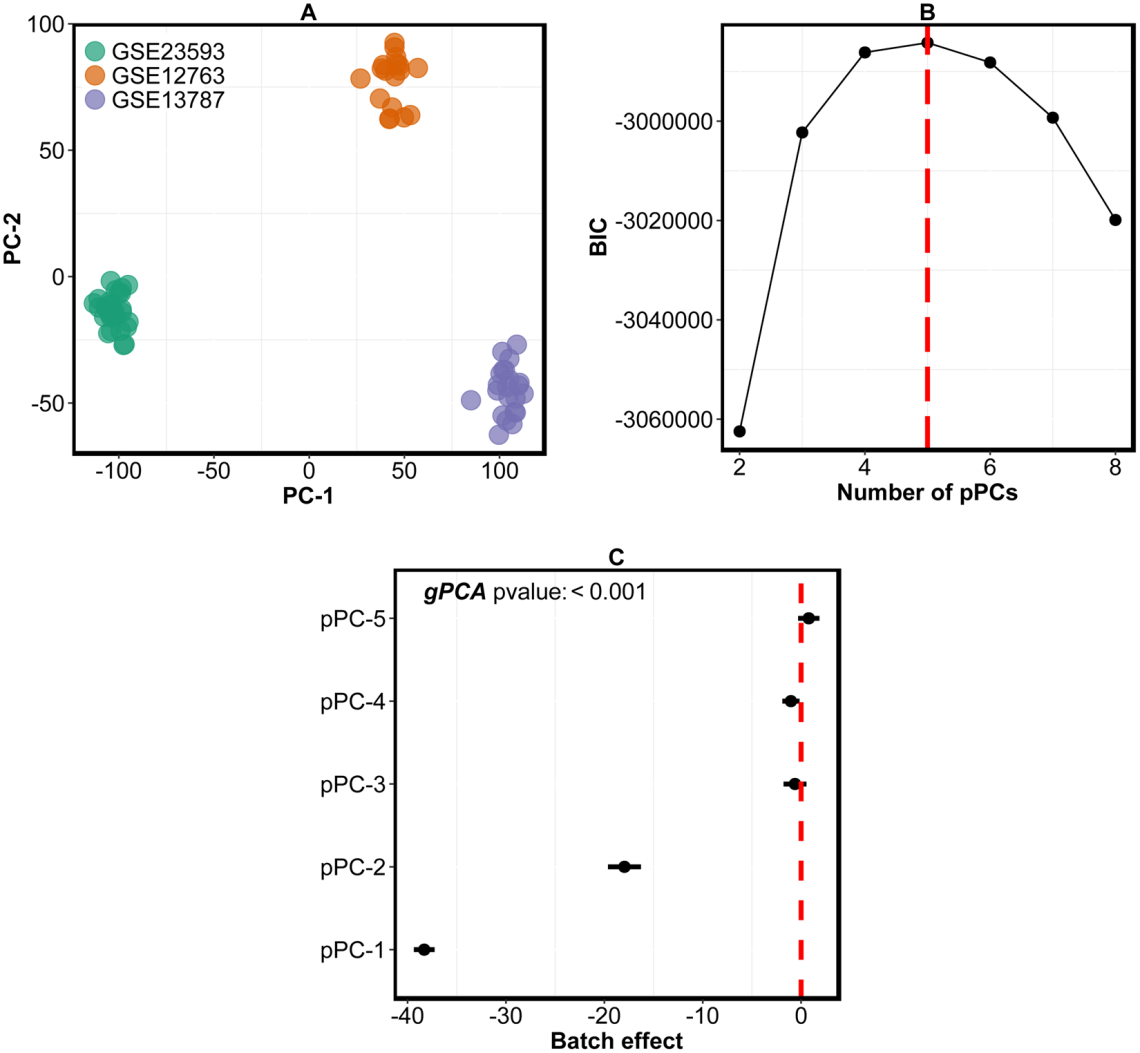
Detection of batch effect in pooled breast cancer gene expression datasets. (**A**) A PCA plot showing clustering of samples according to batches (three breast cancer datasets – GSE23593, GSE13787 and GSE12763). (**B**) BIC values from *findBATCH* showing the optimal number of pPCs for pooled/merged (three) datasets. The higher the BIC value, the better the model. The red dashed vertical line identifies the optimal number of pPCs. (**C**). A forest plot depicting different pPCs from *findBATCH* applied to quantify batch effect before correction.

In this example using breast cancer datasets with batch effect, we demonstrate the utility of *exploBATCH* in detecting, estimating and correcting for batch effect. We also compared results of *exploBATCH* to other commonly used methods – *ComBat* and *gPCA*. Initially, we sought to merge gene expression data (profiled using Affymetrix GeneChip® Human Genome U133 Plus 2.0 Array, 20,155 genes) generated from primary human breast tumors from three different studies of 70 samples (GSE12763^19^, *n* = 30; GSE13787^20^, *n* = 22; and GSE23593^21^, *n* = 18; after microarray quality control; see *Materials and Methods*). It can be clearly seen in Fig. 2A and Supplementary Figure 1 that clustering of samples in the principal subspace (defined by the first two PCs) was exclusively driven by batch effect, which is due to merging data from different sources. However, in situations where batch effect is not the greatest source of variability, PCA may fail to reveal any underlying clustering structure due to batch effect^3^. Consequently, *findBATCH* was applied as a formal statistical test to detect the presence of batch effect in this pooled dataset, by determining the lower dimensional representation of the data affected by batch effect. The BIC plot in Fig. 2B shows that the first five pPCs (with the highest BIC value) explained most of the data variability. The 95% CIs of the estimated regression coefficients associated with batch effect in Fig. 2C (forest plot showing the results of *findBATCH*, which allow us to quantify batch effect and perform formal statistical tests) are used to assess the effect of batch on each of the five pPCs. Fig. 2C shows significant batch effects in pPC1, pPC2 and pPC4 (since their corresponding 95% CIs did not include zero). We further assessed batch effect using another method, *gPCA* ^14^, and it showed a p-value less than 0.001, representing the presence of significant batch effect in these pooled dataset, which is consistent with *findBATCH* analysis (Fig. 2C). However, unlike *findBATCH*, *gPCA* method does not assess the effect of batch on individual PCs. Overall; this establishes the presence of batch effect in the pooled breast cancer dataset.

**Figure 3:**
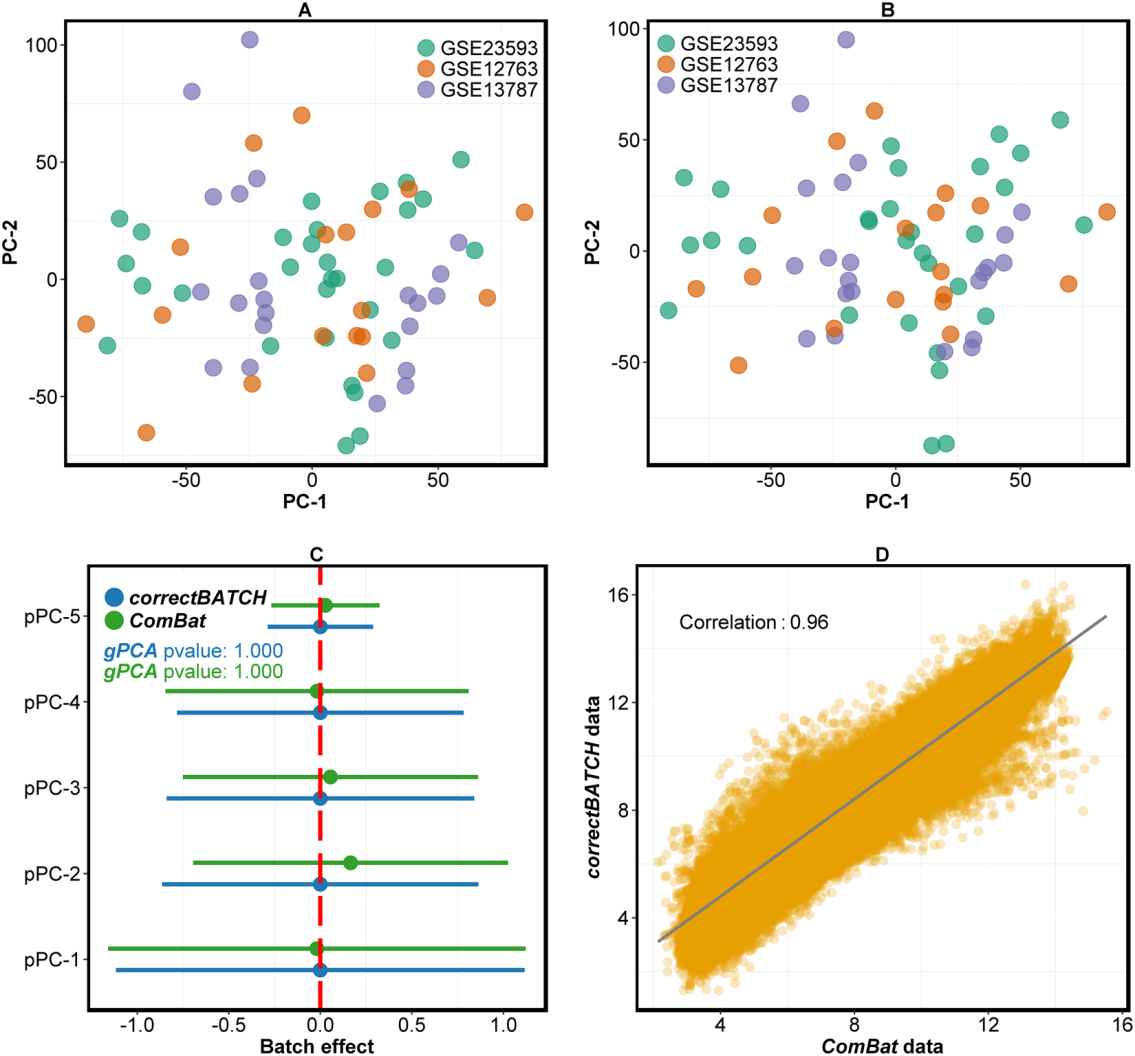
Correction of batch effect in pooled breast cancer gene expression datasets. (**A**,**B**) PCA plots highlight clustering of samples (three breast cancer datasets – GSE23593, GSE13787 and GSE12763) after batch correction using *correctBATCH* (**A**) and *ComBat* **(B)**. (**C**) A forest plot depicting different pPCs from *findBATCH* for assessing batch effect after correction using both *correctBATCH* (blue) or *ComBat* (green). The *gPCA* p-values for the corrected data using *correctBATCH* (blue) or *ComBat* (green) are also shown. (**D**) A plot showing Pearson correlation between *correctBATCH* and *ComBat* batch corrected data.

In order to correct batch effect in this pooled dataset, we applied our *correctBATCH* method, which subtracts the effect of batch in the principal subspace. The performance of *correctBATCH* was compared to *ComBat*, the current standard approach to batch correction^1^, and traditional PCA correction^12, 13^. Although visually inspecting PCA plots in Fig. 3A and B highlighted no batch effect (mixing of samples from different batches) after applying both *correctBATCH* and *ComBat*, formal statistical tests were carried out again using *findBATCH* to assess for any residual batch effect. None of the five pPCs were significantly associated with batch effect after applying the two batch correction methods (95% CIs in Fig. 3C do include zero for all pPCs), confirming the removal of batch effect. We also applied *gPCA* to assess if batch effect has been corrected. A *gPCA* p-value was 1 for both batch correction methods (Fig. 3C), also confirming that batch effect has been removed. The performance of the two batch correction approaches - *correctBATCH* and *ComBat* - in this dataset was generally comparable with a correlation coefficient of 0.96 (Fig. 3D). However, data corrected for batch effect using PCA approach (removing the eigen vectors associated with batch effect^12, 13^) had very low correlation with *correctBATCH* (Pearson correlation coefficient = 0.35) and *ComBat* (Pearson correlation coefficient = 0.26) corrected data, Supplementary Figure 2A and B, respectively. This low correlation could be due to the loss of important biological information when the affected PCs are completely removed in PCA approach. We will demonstrate this in the next example.

Overall, this example demonstrates how *exploBATCH* can be used to: (i) statistically test (instead of simple visual inspection) and quantitate the presence of batch effect using *findBATCH*, and (ii) correct for batch effect using *correctBATCH*.

#### Batch vs. biological effect - merging two colorectal cancer gene expression datasets

When clustering gene expression data containing a mixture of different samples (normal and tumor), tumors typically cluster away from the normal samples; here we refer to this as the “normal/tumor biological effect”. If experiments are not carefully designed, it can be difficult to distinguish biological effects from batch effects. In this example, we demonstrate how *exploBATCH* can be used to disentangle biological variability from batch variability.

Two gene expression datasets (GSE18088^22^ and GSE23878^23^; Affymetrix GeneChip® Human Genome U133 Plus 2.0 Array) consisting of 52 and 58 samples, respectively, (after microarray data analysis quality control; see *Materials and Methods*) were pooled together. Whilst all of the 52 samples from GSE18088 were CRCs, 24 of 58 GSE23878 samples were from normal tissues and the rest were tumor samples (after quality control of the data; see *Materials and Methods*). Here the aim is to assess whether (i) *findBATCH* can distinguish batch effect from biological effect; and (ii) the correction of batch effect using *correctBATCH* retains the normal/tumor biological effect.

**Figure 4:**
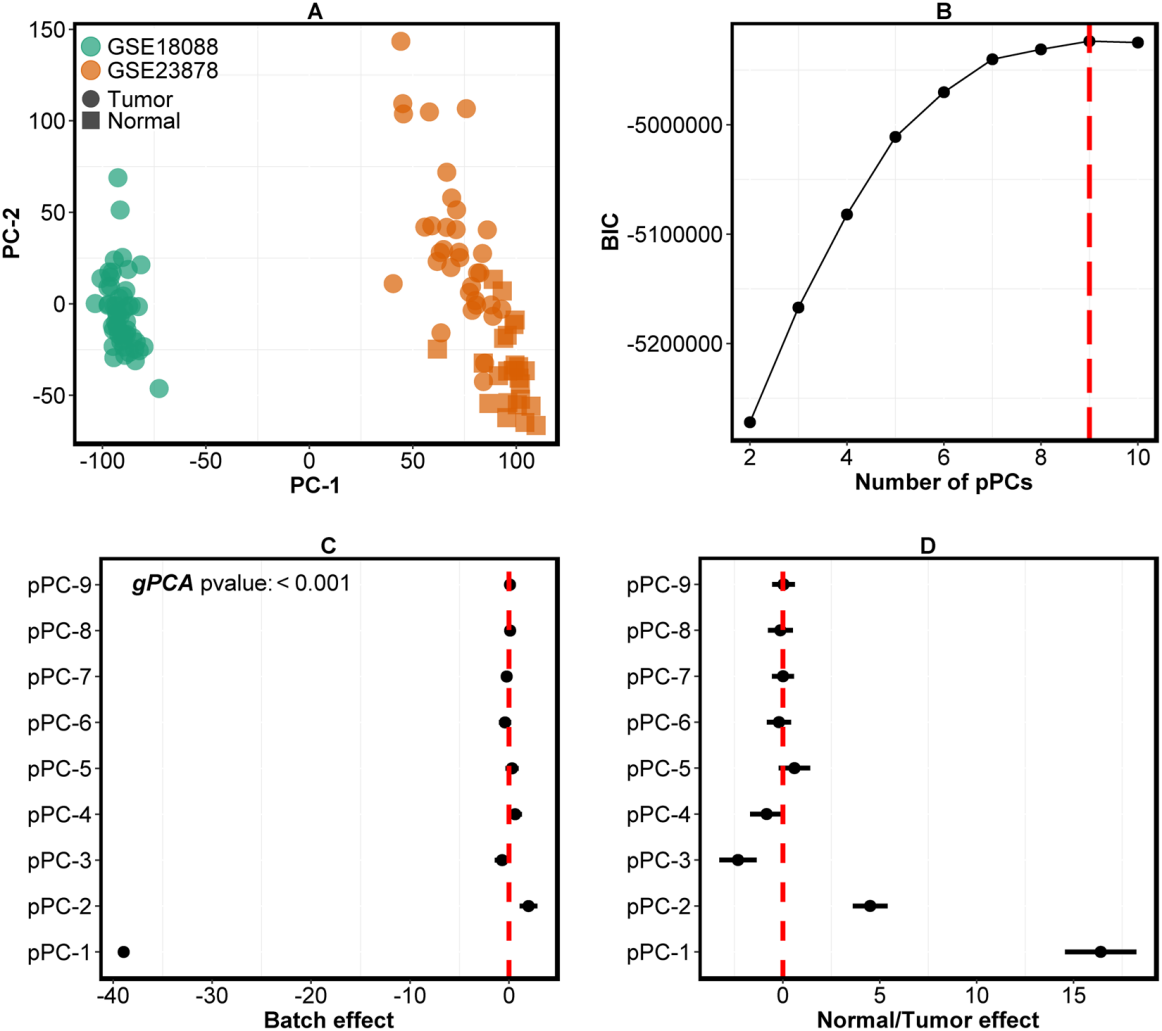
Detection of batch effect in CRC and normal gene expression datasets. (**A**) A PCA plot showing clustering of samples according to batches (the two CRC datasets – GSE18088 and GSE23878) in the principal subspace defined by the first two PCs. The filled squares identify normal samples and the filled circles identify tumors. (**B**) BIC values from *findBATCH* showing the optimal number of pPCs for pooled/merged datasets. The higher the BIC value, the better the model. The red dashed vertical line identifies the optimal number of pPCs to be nine. (**C**,**D**) Forest plots depicting different pPCs from *findBATCH* applied to quantify **(C)** batch and **(D)** normal/biological effect using uncorrected CRC pooled dataset (GSE18088 and GSE23878). pPCs are considered significant only if 95% CIs do not include zero.

Although Fig. 4A and Supplementary Figure 3 highlight batch effect, as samples from GSE23878 dataset clustered away from GSE18088 samples in the first PC, it is difficult to differentiate normal/tumor biological effect from batch effect by visual inspection alone as some normal samples are mixing with tumors. *findBATCH* was applied to detect batch effect in this pooled dataset with two different variables (batch and normal/tumor) as covariates in the PPCCA model. The presence of the normal/tumor variable allowed us to assess if batch correction using either *correctBATCH* or *ComBat* retained biological effect. The optimal number of pPCs for this dataset was nine (BIC plot in Fig. 4B). Whilst the first two of the nine pPCs (pPC1 and pPC2) were significantly associated (95% CIs don’t contain zero) with the batch variable (Fig. 4C), confirming the presence of batch effect in the data, the first three pPCs (pPC1, pPC2 and pPC3) were also associated with the normal/tumor biological effect (Fig. 4D **)**. Hence, batch and biological effects are entangled in the first two PCs, which makes PCA-based batch correction challenging.

**Figure 5:**
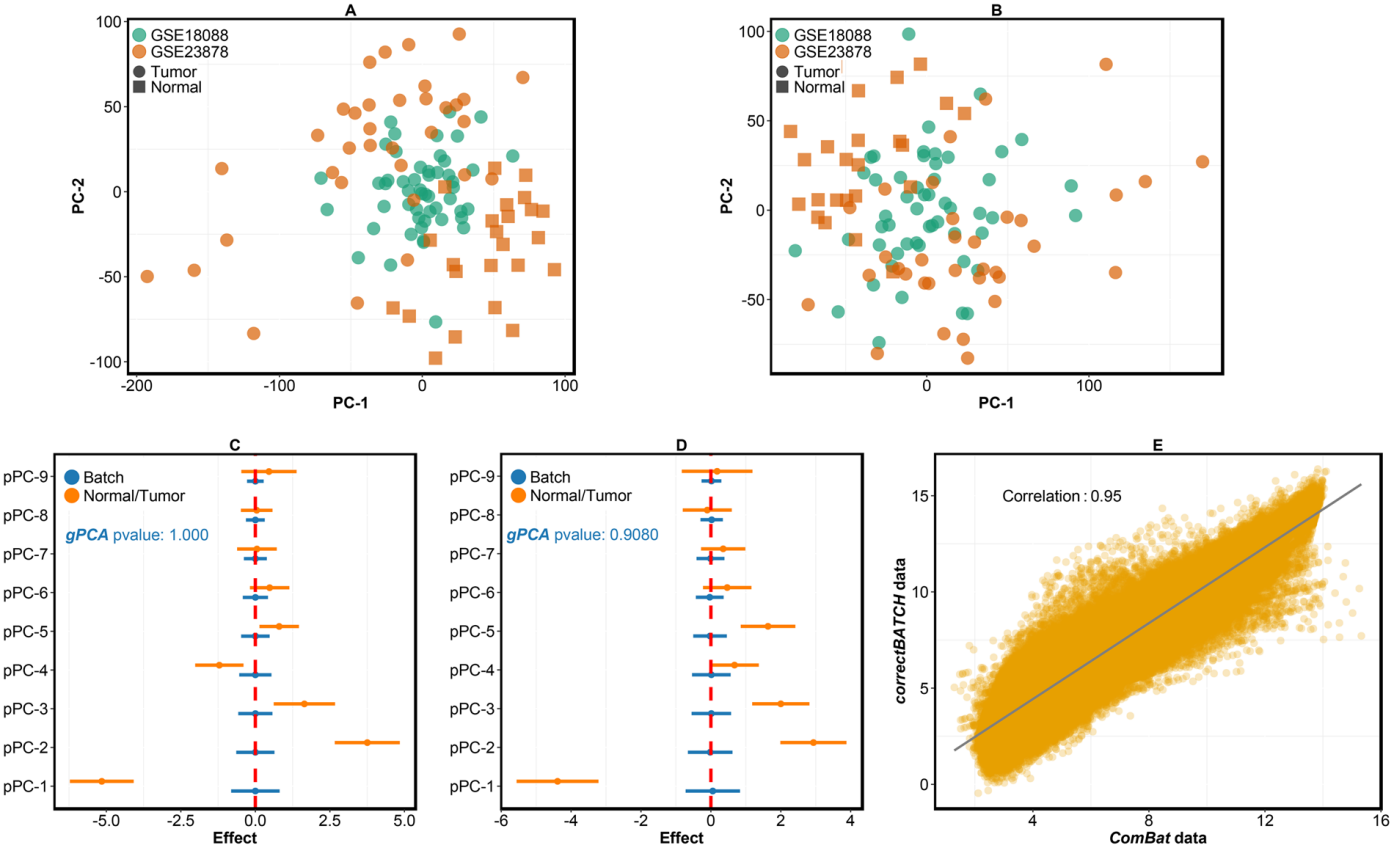
Correction of batch effect in CRC and normal gene expression datasets whilst retaining biological effects. (**A**,**B**) PCA plots showing clustering of samples (the two CRC datasets – GSE18088 and GSE23878) after batch correction using *correctBATCH* **(A)** and *ComBat* **(B)**. (**C**,**D**) Forest plots from *findBATCH* show batch (blue) and normal/tumor biological effect (orange) associated with batch corrected data from **(C)** *correctBATCH* and **(D)** *ComBat*. pPCs are considered significant only if 95% CIs do not include zero. The *gPCA* p-values of 1.000 and 0.908 for *correctBATCH* and *ComBat*, respectively, are shown. (**E**) A plot showing Pearson correlation between batch-corrected data from *correctBATCH* and *ComBat* methods.

In order to efficiently remove the effect of batch whilst retaining biological effects in the data, we applied *correctBATCH*, which removes the effect of batch in each pPC, as well as *ComBat* to assess if the two methods retain biological effect in the data after batch correction. The results of batch correction using these two methods are shown in Fig. 5. Since PCA does not provide a measure to assess the presence of batch effect, it is not easy to conclude from PCA plots in Fig. 5A and B that batch effect has been completely corrected. However, formal statistical tests using *findBATCH* confirmed no significant batch affect (95% CIs in Fig. 5C and D, in blue, do include zero) after applying *correctBATCH* and *ComBat*. Global test of batch effect on all PCs using *gPCA* also confirmed no significant batch effect left in *correctBATCH* and *ComBat* corrected data with p-values of 1.000 and 0.908 (Fig. 5C and D), respectively. Crucially, both batch correction methods managed to retain the normal/tumor biological effect (95% CIs in Fig. 5C and D, in orange, do not include zero). Interestingly, the resolution of normal/tumor biological effect in the data improved after batch correction (Fig. 5C and D; at least one additional pPC was associated with biological effect) compared to that before correction (Fig. 4D **)**. The performance of *correctBATCH* and *ComBat* was comparable with high Pearson correlation coefficient of 0.95 (Fig. 5E).

**Figure 6:**
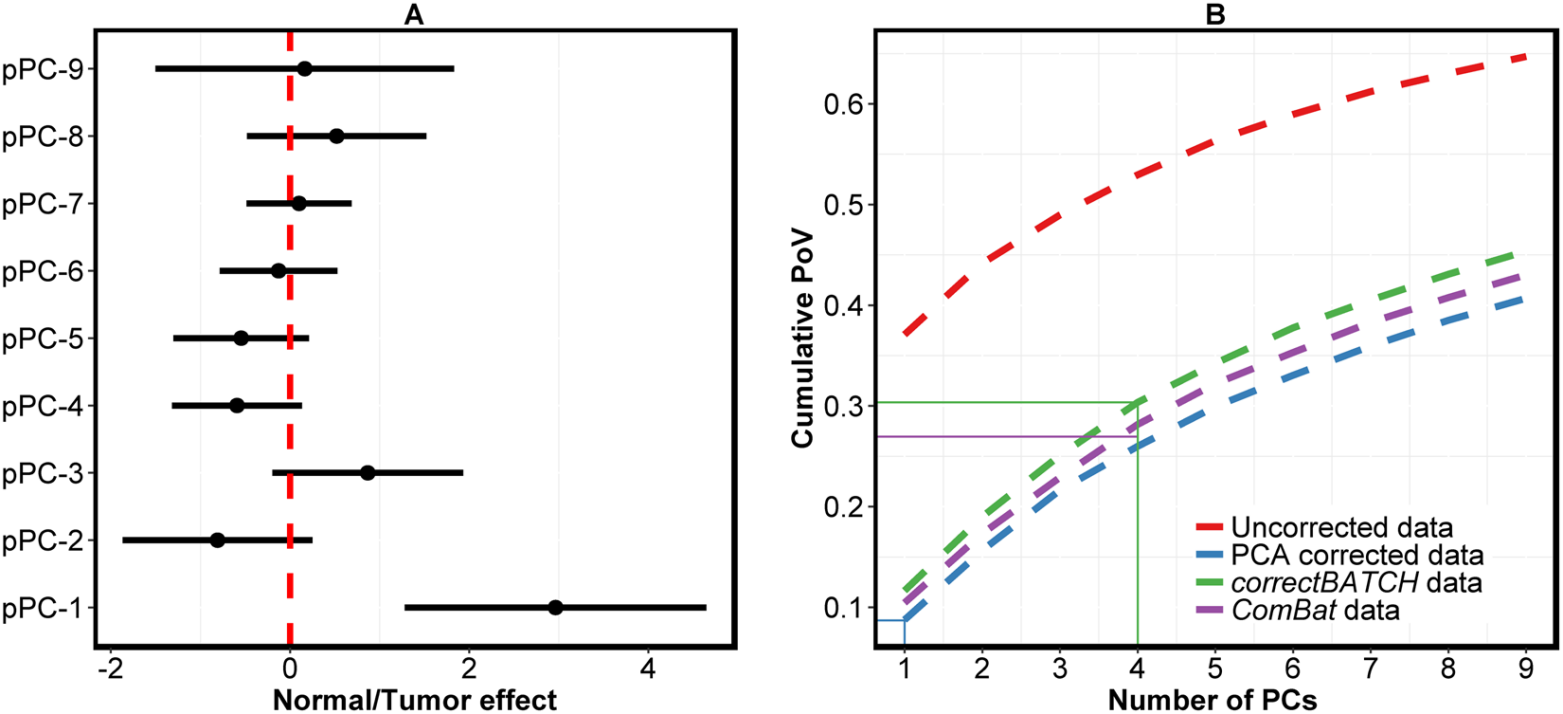
PCA-based batch correction loses biological information. **(A**) A forest plot depicting different pPCs from *findBATCH* applied to assess normal/tumor biological effect after PCA-based batch correction (applied on CRC pooled dataset from - GSE18088 and GSE23878 datasets). pPCs are considered significant only if 95% CIs do not include zero. (**B**) A plot showing the cumulative proportion of variation (PoVs) for the first nine PCs (from PCA) for the uncorrected (red) and corrected (using PCA corrected; blue, *correctBATCH*; green and *ComBat*; violet) CRC pooled data for batch effect. The quadrants highlight the cumulative PoVs for PCs associated with normal/tumor biological effect in PCA (9%; blue), *correctBATCH* (30%; green), and *ComBat* (27%; purple) corrected data.

However, when PCA-based batch correction was applied to the pooled colorectal data, the results did not correlate well with those of *correctBATCH* and *ComBat* (Supplementary Figure 4), with Pearson correlation of 0.36 and 0.23, respectively. We further assessed batch effect on the pooled data using *findBATCH* after PCA-based correction. We observed that only the first probabilistic component (pPC1) from *findBATCH* was associated with the normal/tumor biological effect, as shown in Fig. 6A. In Fig. 6B, this pPC1 only explains less than a tenth (9%) of the total variability in the data corrected for batch effect using PCA. On the other hand, *correctBATCH* and *ComBat*-based batch effect correction of the same data showed additional three pPCs (pPC2, pPC3 and pPC4 in *correctBATCH*; and pPC2, pPC3 and pPC5 in *ComBat;* Fig. 5C and D) associated with a total of 30% and 27% variability of normal/tumor biological effects, respectively. This loss of information in pPC1 of PCA-based batch correction can be explained by the fact that, since batch and biological effects were coupled in pPC1 and pPC2 (as shown in Fig. 4C and D), PCA-based batch correction of simply discarding these two PCs resulted in loss of important normal/tumor biological effect in these two components.

Overall, this example demonstrates that *findBATCH* from *exploBATC*H tool can effectively differentiate batch variability from biological variability to determine and quantitate batch effect in the data. It also detects batch effect even when the results of visual inspection are inconclusive. Moreover, the PPCCA model in *correctBATCH* (from *exploBATC*H tool) allows for correction of batch effect without distorting important biological structures in the data.

## Conclusions

Here, to our knowledge for the first time, we establish a method to evaluate or diagnose batch effect(s) in genomic data at the level of individual PCs. Our method allows for both visual inspection and formal statistical testing of batch effect(s) before and after batch correction. The two methods, *findBATCH* and *correctBATCH*, within the package *exploBATCH* were applied successfully to the two gene expression datasets (breast and colorectal cancer/normal samples) to diagnose and correct for batch effect, respectively. The *correctBATCH* framework allows for removal of batch effect(s) in genomic data without compromising biological effect, provided that the experiments are designed to properly distinguish between batch and biological effects.

## Materials and Methods

### Samples and pre-processing

All the datasets used are publicly available. The three datasets in the first example, GSE12763^19^, GSE13787^20^, and GSE23593^21^, are human breast cancer samples. The gene expression profiles for these breast cancer samples were performed using Affymetrix GeneChip® Human Genome U133 Plus 2.0 Array. The replicate samples were removed from GSE23593 as indicated in GEO Omnibus leading to a total of 18 samples. The samples were pre-processed and normalized using robust multi-array average (*RMA*)^24^ using ***R*** and Bioconductor^25^. One sample (GSM346904) from GSE13787 had normalized unscaled standard error (NUSE; as a part of the *affyPLM* ^26, 27^ package from Bioconductor) median score greater than 1.05, which was removed, leading to a total of 22 samples. GSE12763 had 30 samples. A single probe with highest variation was selected for those genes with multiple probes before merging different datasets. In addition, those probes with gene name not annotated by the HUGO gene nomenclature committee (HGNC)^28^ were removed.

For the second example on colorectal cancer, we chose two gene expression data sets (GSE18088^22^ and GSE23878^23^; Affymetrix GeneChip® Human Genome U133 Plus 2.0 Array) with 53 and 59 samples, respectively. Two samples, one each from GSE18088 and GSE23878, had NUSE median score greater than 1.05, hence were removed from analysis. All 52 samples from the GSE18088 data were primary CRC tumors whilst 24 of 58 GSE23878 samples were normal samples and rest of the samples were matched tumor samples. Again, each of the datasets was pre-processed and normalized using RMA as described above.

### Probabilistic principal component and covariates analysis

Suppose we have measurements, *y*_*i*_ = (*y*_*i*1_…*y*_*ip*)_^T^, taken on a large number of *p* correlated variables (i.e. genes) and corresponding phenotypes (i.e. covariates), *x*_*i*_ = (*x*_*i*1_…*x*_*il*)_^T^, recorded on a sample *i*. PPCCA^18^ can be used to model the relationship between the expression data matrix **Y** = (*y*_*1*_…*y*_*n*)_^T^ and covariates **X** = (*x*_*1*_…*x*_*l*)_^T^ where *n* is the number of samples and *l* is the number of covariates plus an intercept term. High-dimensional data point *y*_*i*_ is modeled as a linear function of the corresponding low-dimensional probabilistic principal component (pPC) score *u*_*i*_ = (*u*_*i1*_…*u*_*iq*)_^T^ (also known as scores in PCA), whilst the pPC score, *u*_*i*_, is modeled as a linear function of covariates *x*_*i*_, plus some unexplained additional sources of variation *ξ*_*i*_ = (*ξ*_*i1*_ *…ξ*_*ip*_)^T^ and ε_*i*_ = (ε_*i1*_…ε_*iq*_) ^T^, respectively, where, *q* «* p*. The PPCCA model can be written as follows,

1 $${\mathop{y}\limits_{\_}}_{i}={\bf{W}}{\mathop{u}\limits_{\_}}_{i}+\mathop{\mu }\limits_{\_}+{\mathop{\xi }\limits_{\_}}_{i},$$

2 $${\mathop{u}\limits_{\_}}_{i}=\beta {\mathop{x}\limits_{\_}}_{i}+{\mathop{\varepsilon }\limits_{\_}}_{i},$$

where **W** is a *p* × *q* loadings matrix, *μ* is a *p* dimensional mean vector of the data and **β** is a *q* × *l* regression coefficients matrix quantifying the relationship between the pPC score *u*_*i*_ and covariates *x*_*i*_. The pPC score, observed errors, and the pPC errors are assumed  to be from multivariate normal distribution (MVN), *u*_*i*_ ~ MVN*q* (**β** *x*_*i*_, **I**), *ξ*_*i*_ ~ MVN*p* (*0*, σ^2^ **I**) and ε_*i*_ ~ MVN*q* (*0*, **I**), where **I** is an identity matrix and σ^2^ is the residual variance. For a more detailed description of the PPCCA model see Nyamundanda *et al*.^18^.

### Explore batch (*exploBATCH*) package

Approaches in *exploBATCH* ***R*** package, to quantitate and correct batch effect, are based on the PPCCA model^18^. Firstly, the PPCCA model is applied to detect batch effect in the dataset. This is achieved by carrying out a formal statistical test to determine if samples are distributed according to batches in the principal subspace defined by the PPCCA model. Since the PPCCA allows for incorporation of covariates in PCA, the batch variable(s) can be tested if it is significantly associated with any of the pPCs from PPCCA using the following test statistic created under the null hypothesis of no batch effect,

3 $${{\rm{\Delta }}}_{bk}=({\beta }_{bk})/SE({\beta }_{bk})$$

where ***β***_*bk*_ is the regression coefficient that quantifies batch effect *b* on *k* ^*th*^ pPC, and *SE* is the corresponding standard error. If this test statistic is significant (5% significance level) for any of the pPCs it confirms the presence of batch effect in the data. Secondly, the effect of batch is removed on those pPCs significantly associated with the batch variable of interest using PPCCA (implemented as *correctBATCH* within *exploBATCH* **R** package) as follows,

4 $${\underline{u}}_{ck}={\underline{u}}_{ak}-{\underline{{\rm{x}}}}_{b}{{\boldsymbol{\beta }}}_{bk}$$

where *u*_*ak*_ and *u*_*ck*_ is a vector of scores of *k* ^*th*^ pPC affected and corrected for batch effect, respectively, whilst *x*_*b*_ is the variable defining batches. Finally, *correctBATCH* recovers the batch effect corrected expression data by using the PPCCA model to predict the observed data but conditioning on the scores, **u** = (**u**_*c*_, **u**_*u*_), where **u**_*c*_ is scores of corrected and **u**_*u*_ is uncorrected pPCs.

In order to improve the speed of matrix multiplications and inversions in *exploBATCH*, Rcpp^29^ packages (such as *RcppArmadillo* ^30^ and *RcppEigen* ^31^), which allow calling **C**++ functions in **R**, were adopted in *exploBATCH* to fit the PPCCA model from *MetabolAnalyze* ^18^ package. **R** packages such as, *foreach* ^32^ and *doParallel* ^33^, were implemented in *exploBATCH* to allow for multi-threading when selecting the optimal number of pPCs and estimating parameter uncertainty. This drastically improved the computational efficiency of *exploBATCH*, compared to *MetabolAnalyze* package that includes PPCCA. The other information and bottlenecks associated with *exploBATCH* implementation are available in the supplementary information (Supplementary Figures 5 and 6).

Other **R** packages in *exploBATCH* include, *SVA* ^34^ for *ComBat*, *STATS* ^25^ for PCA, and *MASS* ^35^ for generating data from a multivariate Gaussian distribution. The *exploBATCH* package is available as an **R** package on github (https://github.com/syspremed/exploBATCH).

## Electronic supplementary material

Supplementary information
