# Supplementary material for: A Novel Statistical Method to Diagnose, Quantify and Correct Batch Effects in Genomic Studies
Source: Sci Rep. 2017 Sep 7;7:10849. doi: 10.1038/s41598-017-11110-6 (PMC5589920; doi:10.1038/s41598-017-11110-6)

## SUPPLEMENTARY INFORMATION

**Article**

# A Novel Statistical Method to Diagnose, Quantify and Correct Batch Effects in Genomic Studies

**Gift Nyamundanda[1,2], Pawan Poudel[1], Yatish Patil[1,2] and Anguraj Sadanandam[1,2,*]**

[1] Division of Molecular Pathology, The Institute of Cancer Research, London, United Kingdom

[2] Centre for Molecular Pathology, Royal Marsden Hospital, London, United Kingdom

* - Correspondence to anguraj.sadanandam@icr.ac.uk

**Keywords:** batch detection, batch quantitation, statistical measures, microarrays, RNAseq, principal component analysis, probabilistic principal components and covariates analysis, batch correction, batch effect, cancer.

## Benchmarking *exploBATCH* package

Memory usage and running time for *exploBATCH* was assessed using data in the first example (in the main text), consisting of 20,155 genes and 70 breast cancer samples. High performance computer (E5-2670 Xeon, two central processing units (CPUs) of 2.6 gigahertz) with 16 cores was used to run *exploBATCH* on several gene expression datasets extracted from the breast cancer data with 70 samples, by selecting genes using several standard deviations between 0.7 and 2.6. **Supplementary Figure 5A** shows that *exploBATCH* runtime and memory usage for this example with 70 samples and number of genes ranging from 100 to 20,155 was between 0.22 and 531 minutes and 0.2 and 92 gigabytes (GB), respectively.

Also, simulations were carried out by varying the number of samples ($n$) and the number of features ($p$), to assess the computational complexity and runtime of *exploBATCH*. Initially, the batch variable, with two batches $b_1$ and $b_2$, was simulated from a binomial distribution with probability of 0.5 using *rbinom*[1] function in ***R***. Then the expression data was generated from a multivariate Gaussian distribution, using *mvrnorm* function (MASS[2] R package), allowing the two batches to have different mean and different covariance structure. The average expression of probes in batch $b_1$ was set at 100 with variance two and correlation of 0.8. The second batch, $b_2$, was allowed to have lower average expression, set at 50, but with unit variance and correlation between probes of 0.1.

iMac (Apple Inc.) with Macintosh OS X EI Captain (version 10.11) and one CPU with four cores each of 8 GB of random access memory (RAM) was used to assess *exploBATCH* runtimes on the simulated data. **Supplementary Figure 5B** shows that running time of *exploBATCH* mainly depends on the number of probes and samples. *exploBATCH* will take additional time depending on the number of probes and samples. Since Jack-knifing[3,4] approach is used to estimate parameter uncertainty in *exploBATCH*, running times for sample sizes of more than 200 takes an exponential increase after 750 probes.

**Random noise is minimal in *exploBATCH***

In general, unexplained variations within data are modelled as random noise (randomness) by any statistical model. We evaluated the randomness arising from the *exploBATCH* package and its effect on batch corrected data. The randomness can be quantified by the residual noise variance (estimated by the average of the eigenvalues of the discarded principal components). When this noise variance increases, the randomness also increases. So far from the experiments we have done repeatedly running *exploBATCH* on the same dataset 30 times, the correlation between different runs of *exploBATCH* was at least 0.9999 (**Supplementary Figure 6)**. Hence, we expect minimal randomness and consequently less variation in batch corrected data from multiple runs of PPCCA within the *exploBATCH* package. However, using "set.seed"[5] in **R**, we further removed the randomness and variation in data, and the results from multiple runs of *exploBATCH* are reproducible provided the data is same.

## References

1. Kachitvichyanukul, V. & Schmeiser, B. W. Binomial random variate generation. *Commun. ACM* **31,** 216–222 (1988).
2. Ripley, W. N. V. & B. D. Package ‘ MASS ’. *Mod. Appl. Stat. with S* (2002).
3. Efron, B. Bootstrap Methods: Another Look at the Jackknife. *Ann. Stat.* **7,** 1–26 (1979).
4. Nyamundanda, G., Brennan, L. & Gormley, I. Probabilistic principal component analysis for metabolomic data. *BMC Bioinformatics* **11,** 571 (2010).
5. Ahrens, J. H. & Dieter, U. Extensions of Forsythe’s method for random sampling from the normal distribution. *Math. Comput.* **27,** 927–927 (1973).

## SUPPLEMENTARY FIGURE LEGENDS

**Supplementary Figure 1: Pairwise PCA plots of the pooled breast cancer datasets before batch correction.** The first five PCs were compared.

**Supplementary Figure 2: Comparing PCA-based batch correction to the other two batch correction methods using the pooled breast cancer dataset. A-B.** PCA-based batch correction was compared with *correctBATCH* and *ComBat* using pooled breast cancer data. Pearson correlation between

PCA batch corrected data and **(A)** *correctBATCH* or **(B)** *ComBat* batch corrected data.

**Supplementary Figure 3: Pairwise PCA plots of the pooled dataset of colorectal cancer and normal samples.** The first nine PCs were compared.

**Supplementary Figure 4: Comparing PCA-based batch correction to the other two batch correction methods using the pooled colorectal cancer dataset. A-B.** PCA-based batch correction was compared with *correctBATCH* and *ComBat* using pooled colorectal cancer dataset. Pearson correlation between PCA batch corrected data and **(A)** *correctBATCH* or **(B)** *ComBat* corrected data.

**Supplementary Figure 5: Assessing runtime and memory usage of *exploBATCH* tool. A.** Run time profiles for gene expression datasets with 70-breast cancer samples but different sets of genes selected using standard deviation (SD). **B.** Run time profiles of several simulated data varying the number of samples and probes as represented.

**Supplementary Figure 6: Assessing randomness of running *exploBATCH* at different times.** Pairwise comparison of exploBATCH corrected simulated data from 30 different runs using Pearson correlation.

**Supplementary Figure 1**

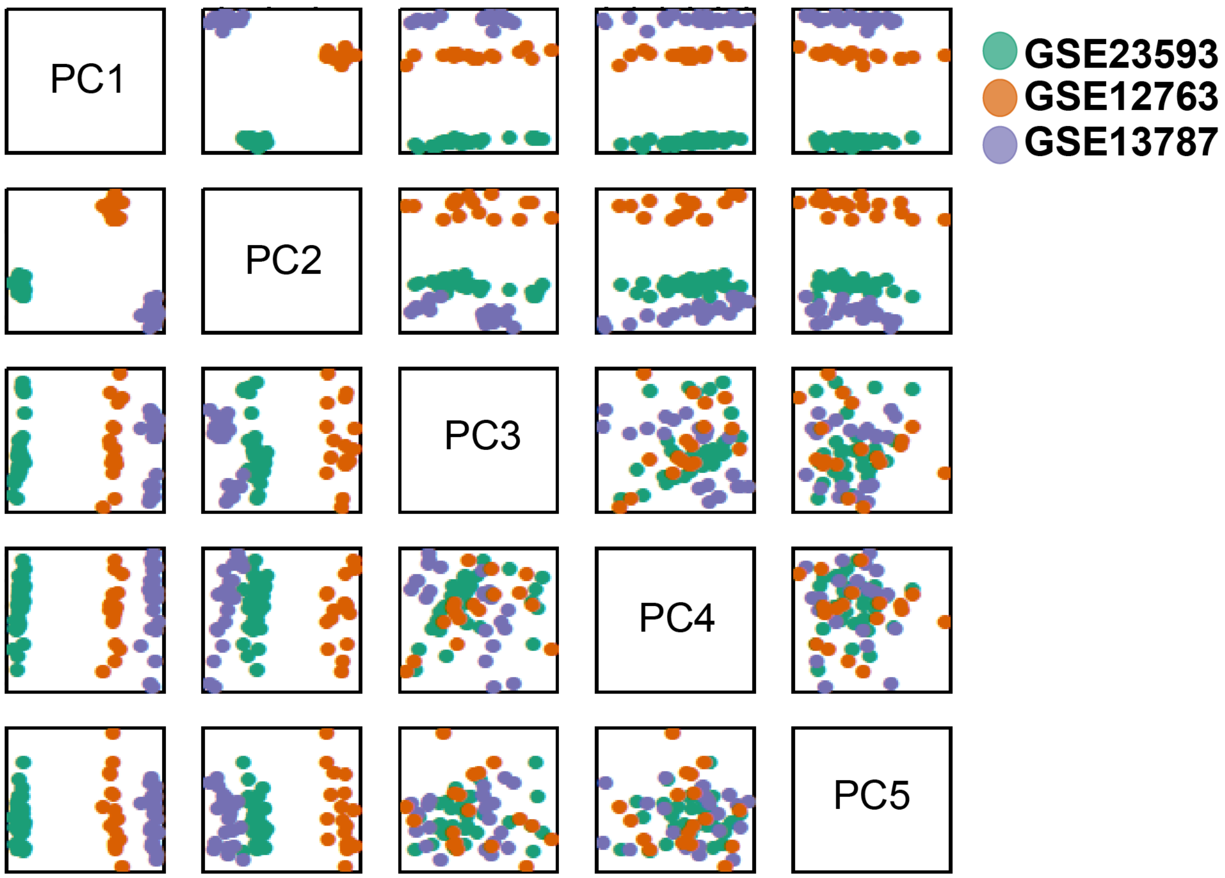

Supplementary Figure 2

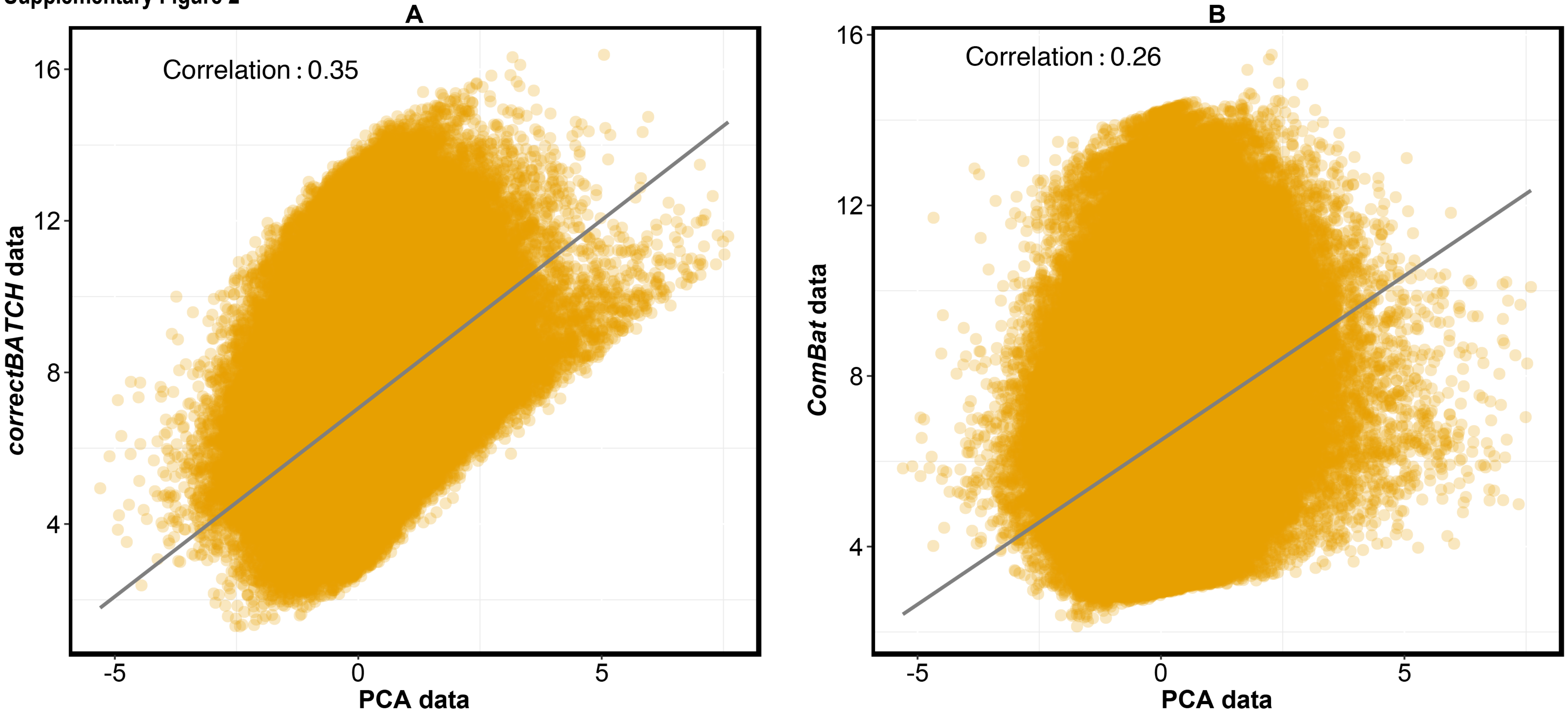

## Supplementary Figure 3

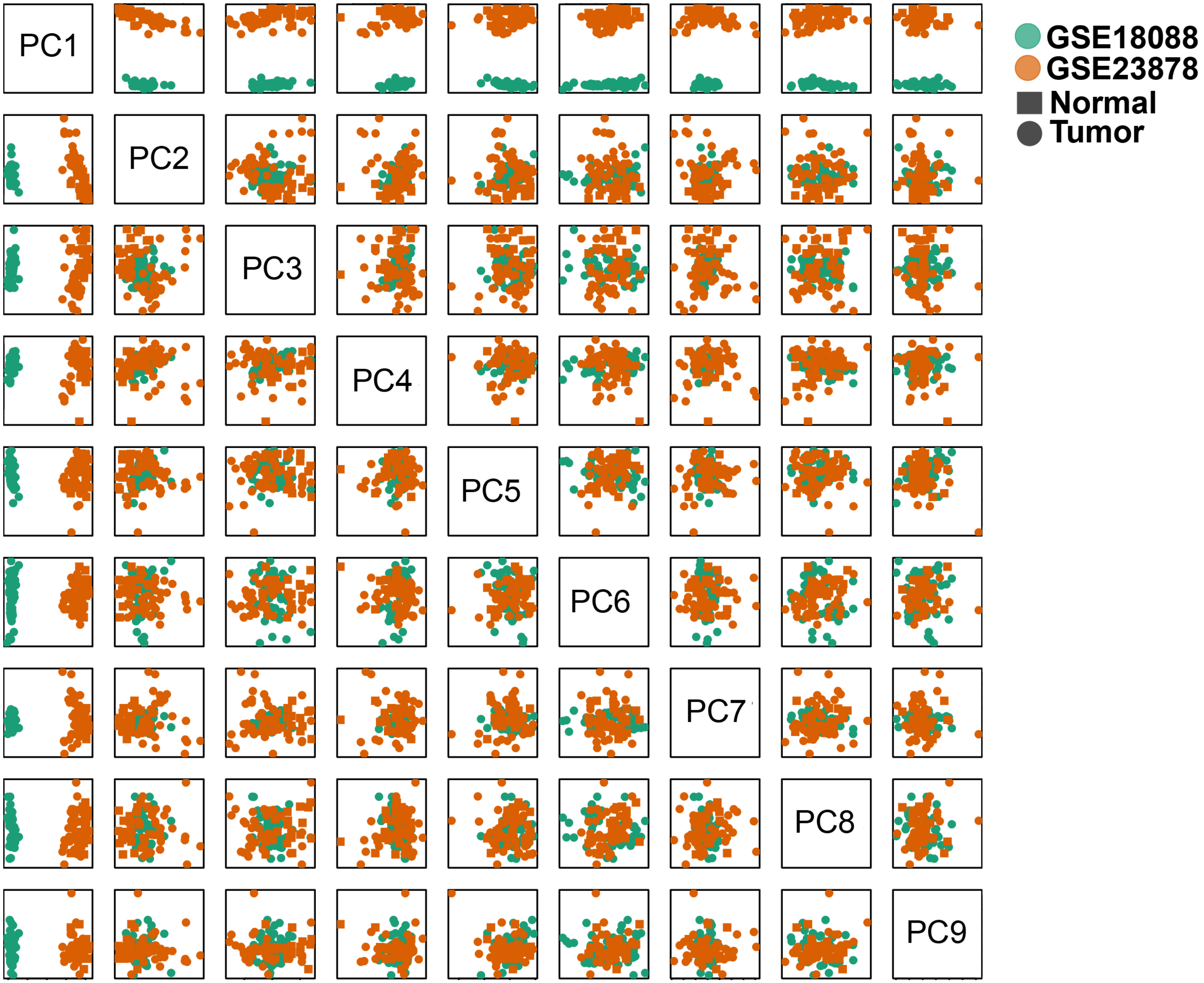

**Supplementary Figure 4**

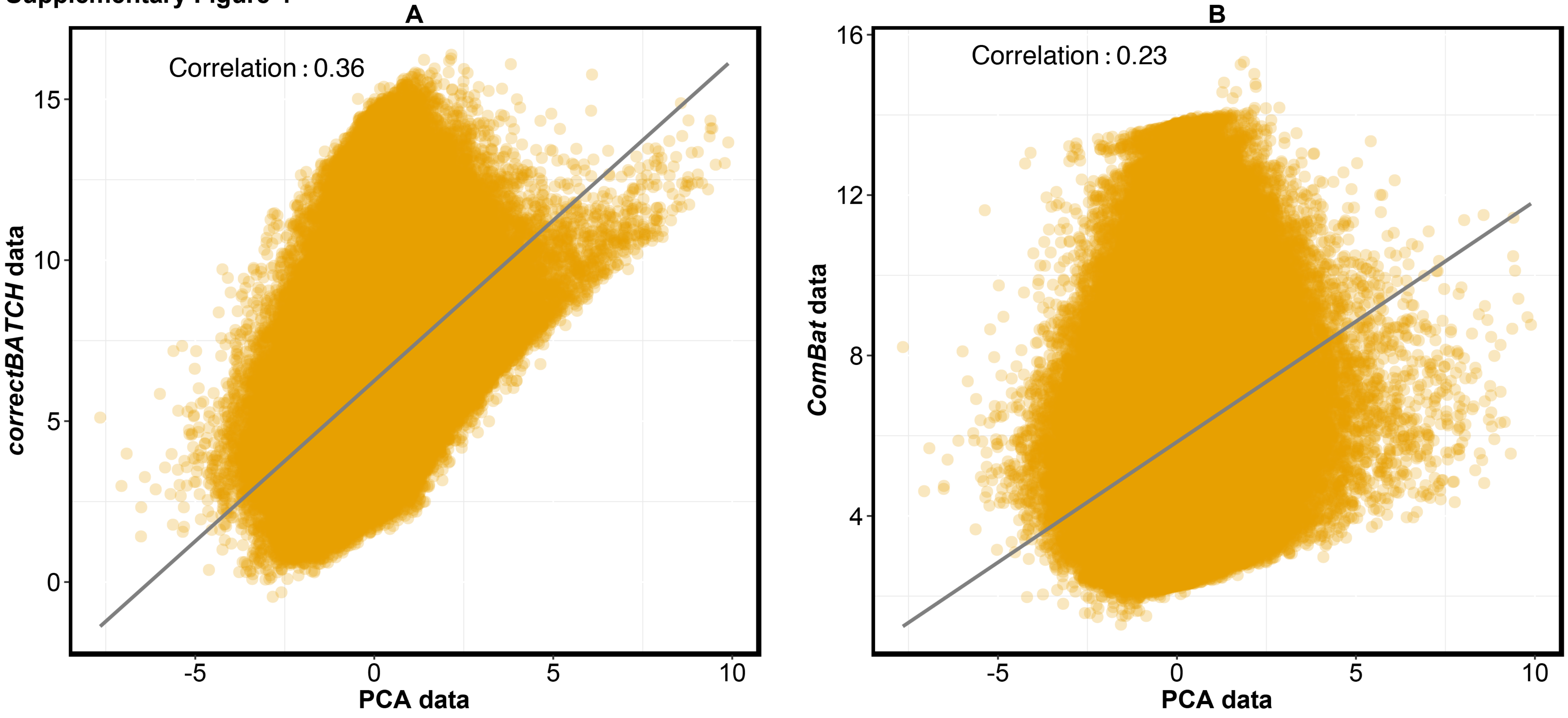

**Supplementary Figure 5**

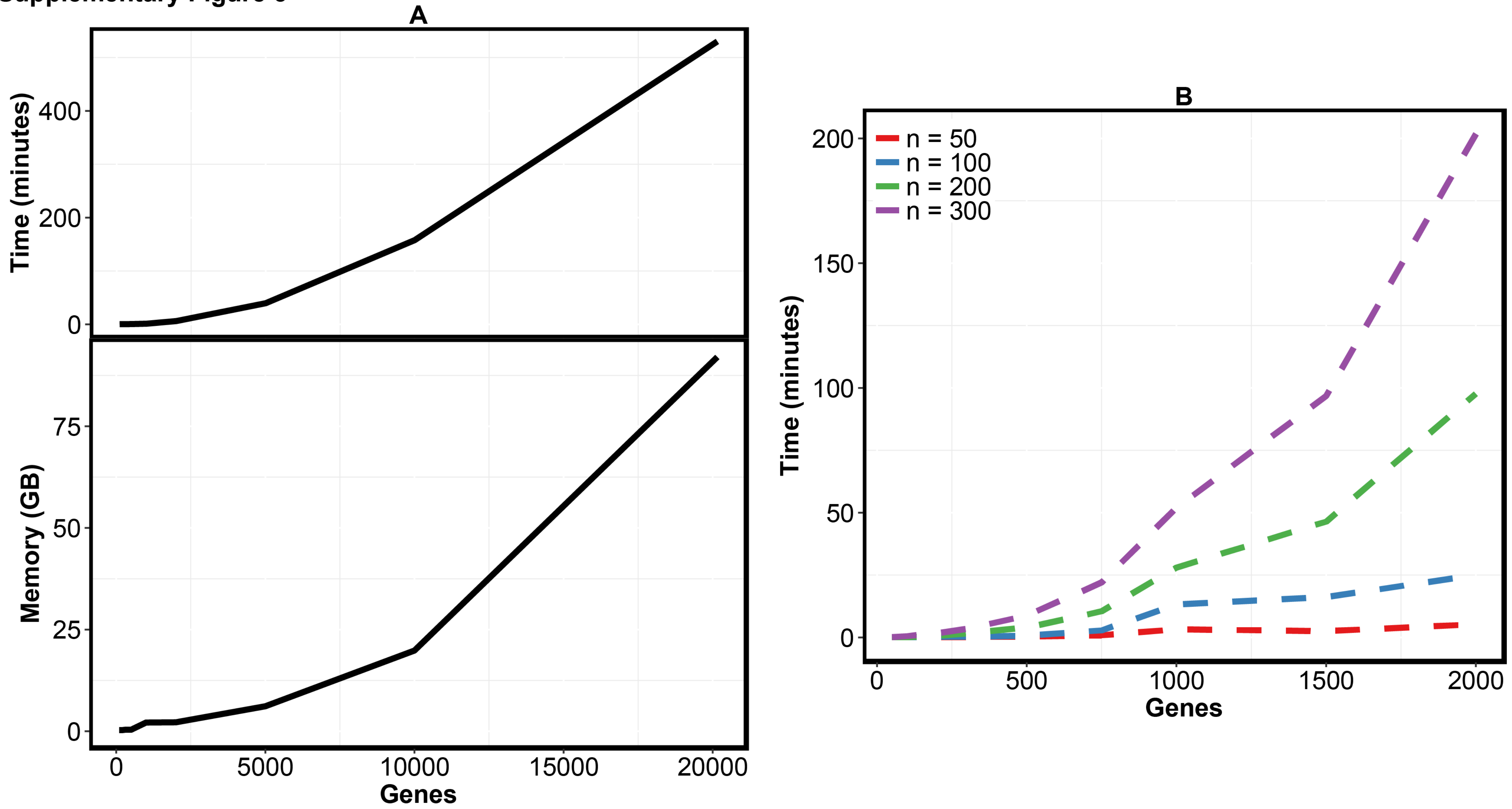

**Supplementary Figure 6**

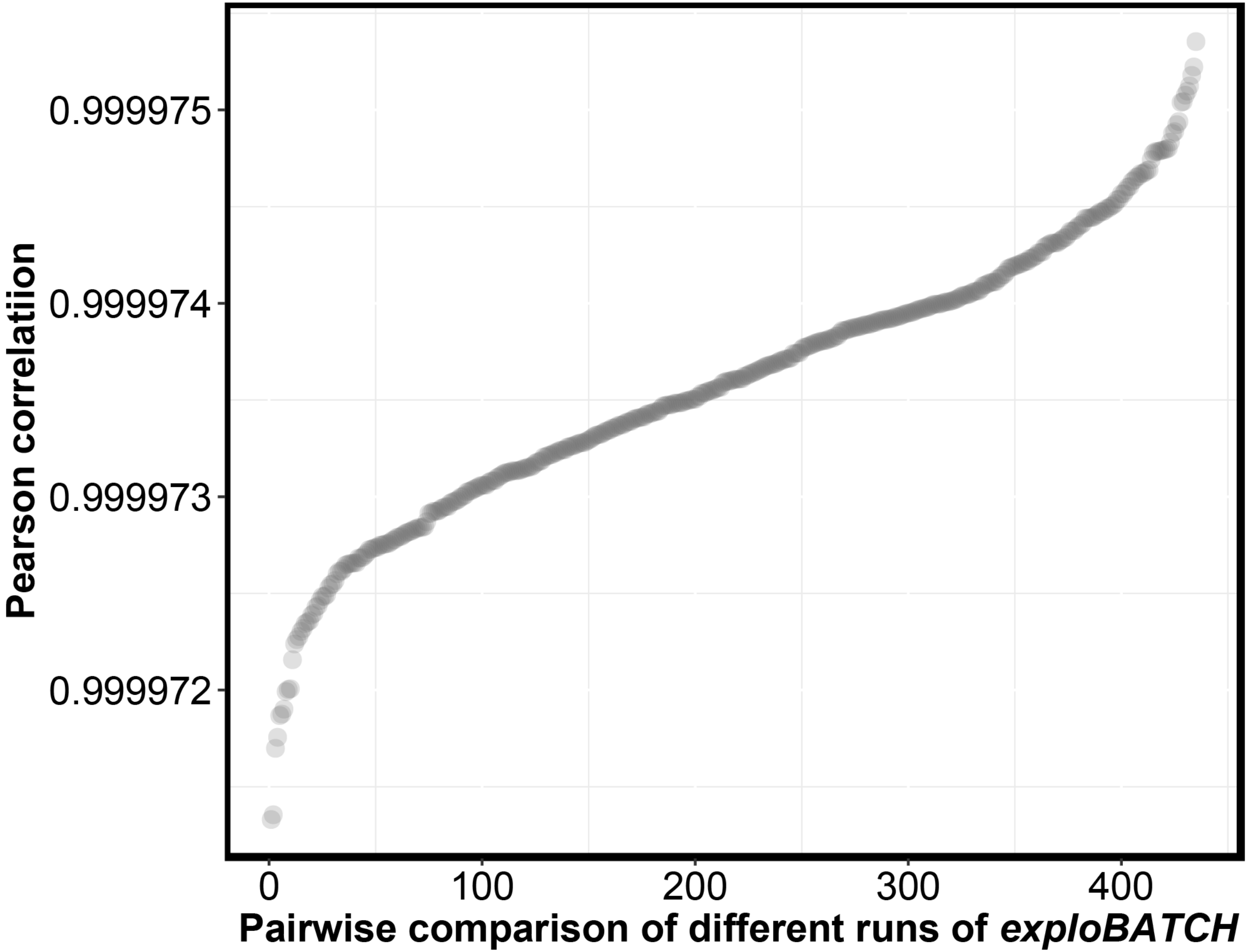

Supplement: Supplementary file 1 — Supplementary information [file 41598_2017_11110_MOESM1_ESM.pdf]
